# Effects of Ambient Gases on the Electrical Performance of Solution-Processed C8-BTBT Thin-Film Transistors

**DOI:** 10.1186/s11671-019-3007-x

**Published:** 2019-05-21

**Authors:** Jiaying Mai, Naiwei Tang, Waner He, Zhengmiao Zou, Chunlai Luo, Aihua Zhang, Zhen Fan, Sujuan Wu, Min Zeng, Jinwei Gao, Guofu Zhou, Xubing Lu, J-M Liu

**Affiliations:** 1 Institute for Advanced Materials, South China Academy of Advanced Optoelectronics, and Guangdong Provincial Key Laboratory of Optical Information Materials and Technology, South China Normal University, Guangzhou, 510006 China; 2 Guangdong Provincial Laboratory of Quantum Engineering and Quantum Materials and Institute for Advanced Materials, South China Academy of Advanced Optoelectronics, South China Normal University, Guangzhou, 510006 China; 3 Guangdong Provincial Key Laboratory of Optical Information Materials and Technology and Institute of Electronic Paper Displays, South China Academy of Advanced Optoelectronics, South China Normal University, Guangzhou, 510006 China; 4 Laboratory of Solid State Microstructures and Innovation Center of Advanced Microstructures, Nanjing University, Nanjing, 210093 China

**Keywords:** Solution process, C8-BTBT, Thin-film transistors, Air stability, Ambient gases

## Abstract

We performed a systematic study of the influence of environmental conditions on the electrical performance characteristics of solution-processed 2,7-dioctyl [1] benzothieno[3,2-b][1]-benzothiophene (C8-BTBT) thin-film transistors (TFTs). Four environmental exposure conditions were considered: high vacuum (HV), O_2_, N_2_, and air. The devices exposed to O_2_ and N_2_ for 2 h performed in a manner similar to that of the device kept in HV. However, the device exposed to air for 2 h exhibited significantly better electrical properties than its counterparts. The average and highest carrier mobility of the 70 air-exposed C8-BTBT TFTs were 4.82 and 8.07 cm^2^V^-1^s^-1^, respectively. This can be compared to 2.76 cm^2^V^-1^s^-1^ and 4.70 cm^2^V^-1^s^-1^, respectively, for the 70 devices kept in HV. Furthermore, device air stability was investigated. The electrical performance of C8-BTBT TFTs degrades after long periods of air exposure. Our work improves knowledge of charge transport behavior and mechanisms in C8-BTBT OTFTs. It also provides ideas that may help to improve device electrical performance further.

## Introduction

Due to the advantages of low deposition temperature, high mechanical flexibility, low cost, and large area production, organic semiconductor materials have recently been widely investigated for various electronic device applications such as organic light-emitting diodes, organic photovoltaic devices, and organic field-effect transistors [1–4]. Organic semiconductors can be divided into two main categories: conjugated polymers and small molecule organic semiconductors [3]. Compared with conjugated polymers, small molecule organic semiconductors offer high degrees of ordering, stacking density, and material purity. These advantages facilitate the fabrication of high-performance devices [5–8]. C8-BTBT is a representative small molecule organic semiconductor material [5]. Extensive research has been performed to study its charge transport mechanisms [9], low-cost fabrication methods [10, 11], growth and microstructure formation on various substrates [12–14], metal/semiconductor contact characteristics [15, 16], and strategies to increase its carrier mobility [11, 17–19]. Thus far, there is no systematic study on the impact of ambient gases on the electrical performance of C8-BTBT-based devices. On the one hand, environmentally induced changes to the electrical performance characteristics of such organic devices are a critical problem that must be solved to provide stable operation for future commercial applications. On the other hand, such effects imply the potential for use of C8-BTBT-based devices as gas sensors.

In this study, C8-BTBT organic semiconductor films were fabricated via solution processing. The electrical properties of the C8-BTBT-based OTFTs were investigated in various ambient gases. The C8-BTBT OTFTs exhibited their highest carrier mobilities (~ 8 cm^2^V^-1^s^-1^) after exposure to air for 2 h. This is assumed to be closely related to the moisture in the air. The study also revealed that changes in the internal molecular structure play important roles in the electrical performance of the OTFTs. The present work not only deepened the understanding on the charge transport mechanisms and structural changes in C8-BTBT films but also provides new ideas to further improve their electrical performances.

## Methods

### C8-BTBT Deposition and OTFT Device Fabrication

A highly doped p-type silicon (100) wafer with a 50 nm thermally oxidized SiO_2_ layer was used as the substrate for organic thin-film transistor preparation. The Si wafer was used as the bottom gate electrode, and the SiO_2_ layer acted as the gate insulator. The substrates were cleaned with acetone, isopropanol, and deionized water for 5 min each using an ultrasonic cleaner. To ensure that the substrate surfaces were clean and dry, the substrates were dried on a hot plate in air for 15 min at 120 °C. In order to change the surface hydrophobicity, all the samples received a UV-ozone treatment for 1 min. This treatment time was chosen based on our previous results [10]. In a previous study, a C8-BTBT OTFT exposed to 1 min of UV surface treatment exhibited better electrical performance than those exposed to other UV treatment durations or non-UV treatment. The organic semiconductor layer was made from high-purity C8-BTBT (≥ 99%) (Sigma-Aldrich) and PMMA (Aladdin) dissolved in chlorobenzene. The solution (0.5 wt% C8-BTBT and 0.5 wt% PMMA) was spin-coated onto 50 nm SiO_2_ covered p++ substrate (2000 rpm for 40 s). Each spin-coating cycle produced one 45 nm layer of C8-BTBT film. After annealing at 60 °C for 2 h in air, MoO_3_ (5 nm) was deposited via thermal evaporation through a metal mask. This buffer layer was designed to reduce the contact barrier between the Au electrode and C8-BTBT semiconductor and to improve charge injection. Finally, Au source and drain electrodes (40 nm) were fabricated via thermal evaporation using the same MoO_3_ shadow mask. The resulting transistor devices had various channel lengths that ranged from 50 to 350 μm, but the same channel width of 1200 μm.

### Material and Device Characterization

An Agilent B1500A semiconductor device analyzer was used to measure device electrical performance. Surface morphologies and roughnesses were observed via tapping mode atomic force microscopy (Asylum Research). Raman spectroscopy characterizations were performed using a Renishaw in Via Raman Microscope. The C8-BTBT layer thickness was measured using an ellipsometer.

Before their electrical performance measurements, the devices were stored in specific environmental conditions (high vacuum, N_2_, O_2_, air) for 2 h so that they would be fully exposed to the desired gases. For convenience, the devices exposed to high vacuum (1.3 × 10^−5^ Torr), N_2_, O_2_, and air will be referred to as the HV, N_2_, O_2_, and air devices, respectively. For each environmental condition or ambient gas, 70 devices were measured in order to produce reliable and statistically meaningful electrical performance results. In addition, the electrical performance of one sample was monitored as a function of the air exposure time to study its stability in air.

## Results and Discussion

**Fig. 1:**
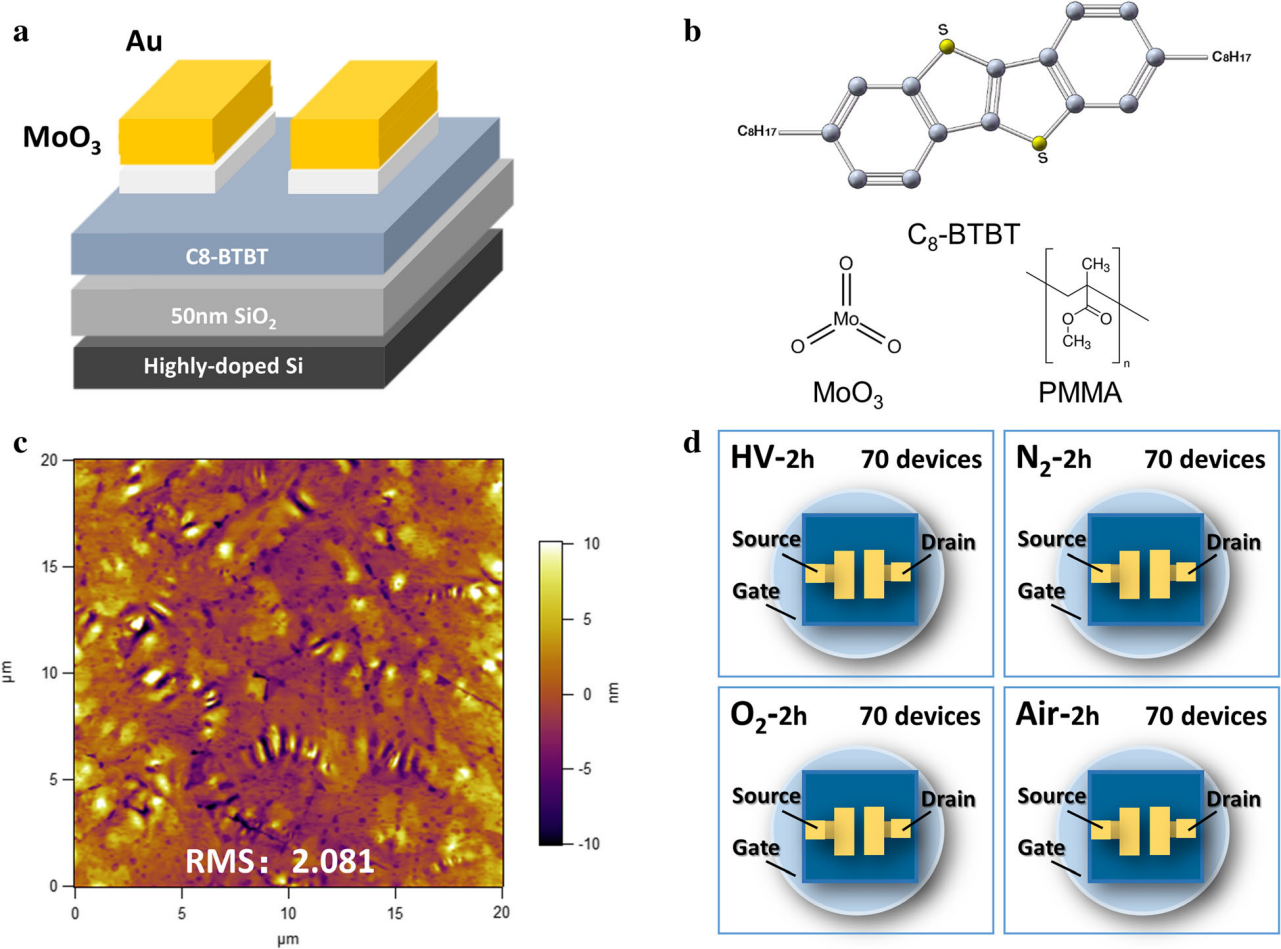
(Color online) (**a**) A schematic diagram of the device structure. (**b**) The molecular structures of the C8-BTBT, molybdenum oxide, and PMMA used in the experiment. (**c**) AFM surface morphology image of the C8-BTBT film indicating a small RMS value of 2.08 nm. (**d**) Test procedures used to measure the electrical performance characteristics of 70 units of each device type (high vacuum, nitrogen atmosphere, oxygen atmosphere, and air atmosphere)

The cross-sectional structure of the OTFT device is shown schematically in Fig. 1a. From bottom to top, it consists of a highly doped Si substrate, 50 nm of silicon oxide, 45 nm of C8-BTBT film, and Au(40 nm)/MoO_3_(5 nm) electrodes. Au/MoO_3_ source/drain electrodes were used to reduce the contact barrier between the Au electrodes and C8-BTBT, which can help to increase the charge injection efficiency and produce high-mobility devices [10]. Figure 1b shows the molecular structures of C8-BTBT, MoO_3_, and PMMA. It should be noticed that PMMA was added into C8-BTBT to make a mixed solution in our work. Blending a polymer into a small molecule organic semiconductor is a common method to improve electrical performances of an organic semiconductor. It helps to form a smooth, continuous semiconductor film. In addition, differences in mass induce vertical phase separation, which is expected to reduce the number of surface traps in the semiconductor [19]. An AFM surface morphology image of the C8-BTBT thin film is shown in Fig. 1c. It indicates large grain-size, good surface continuity, and a smooth surface morphology (RMS value 2.081 nm). Figure 1d shows schematic diagrams of the test procedures used with samples that had been exposed to HV, nitrogen, oxygen, and air. For each ambient gas, 70 devices were measured after 2 h of exposure.

**Fig. 2:**
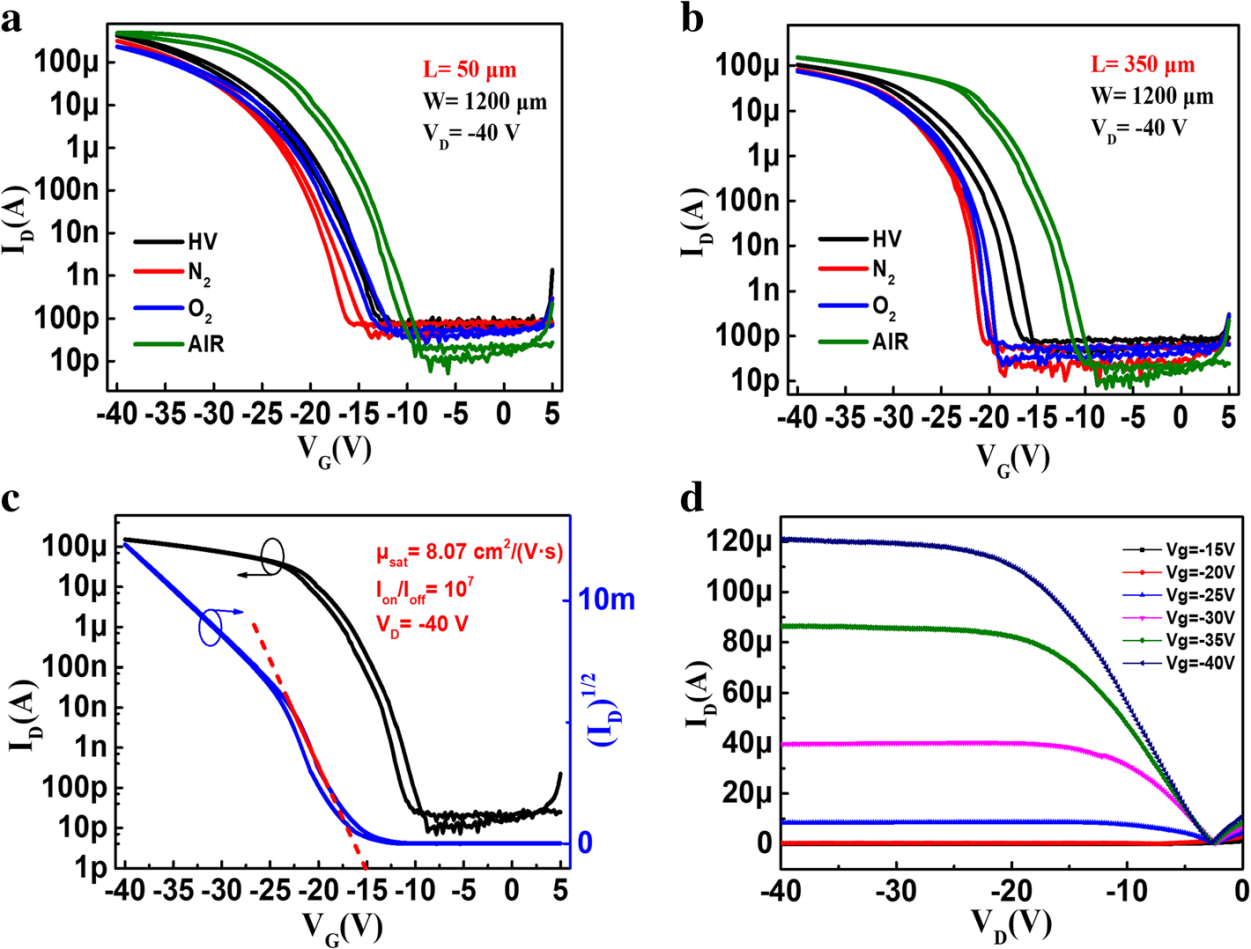
(Color online) Typical transfer characteristics of transistors after exposure to various environmental conditions: 50 μm (**a**) and 350 μm (**b**) channel lengths. Typical transfer characteristics (**c**) and output characteristics (**d**) of devices with mobilities of 8.07 cm^2^ (V s)^−1^, *I*_on_/*I*_off_ ratios of 10^7^, and 350-μm-long channels

To clarify how the different ambient gases affect device electrical performance, the transfer characteristics of these four device types were compared. Figure 2a and 2b show typical drain current-gate voltage (*I*_D_-*V*_G_) curves of short channel (*L* = 50 μm) and long channel (*L* = 350 μm) devices, respectively. All of the devices have the same channel width of 1200 μm and were measured using the same − 40 V drain voltage. No significant hysteresis loops are observed regardless of gas exposure or channel length. An obvious decrease in the off-state drain current (*I*_off_) and increase in the on-state drain current (*I*_on_) are observed for the device exposed to air. Its on/off drain current ratio is as high as 10^7^, while those of HV devices, O_2_ devices, and N_2_ devices are 10^6^. In addition, the air device exhibits carrier mobility that is almost twice as high as those of the other devices and a *V*_TH_ that is 5 to 8 V lower. The results shown in Fig. 2a and 2b demonstrate that the device exposed to air for 2 h exhibits better electrical properties than those exposed to other ambient gases. Typical transfer (*V*_D_ = − 40 V) and output characteristics of air devices with a channel length of 350 μm are shown in Fig. 2c and 2d, respectively. These figures show the outstanding electrical performance characteristics of the solution processed C8-BTBT transistors. A well-saturated *I*_D_-*V*_G_ curve, large *I*_on_/*I*_off_ of 10^7^, and high carrier mobility of 8.07 cm^2^V^-1^s^-1^ are observed. The small hysteresis loop shown in Fig. 2c indicates that an imperfect interface is present between the C8-BTBT and SiO_2_. The non-linear *I*_D_-*V*_D_ curves at low drain voltage shown in Fig. 2d indicate that the potential barrier at the contact interface is still not low enough for ohmic conduction, despite the use of a MoO_3_ layer to reduce the interfacial barrier between the S/D electrodes and semiconductor. The electrical performance of the air device can be further improved via future interface optimization.

**Fig. 3:**
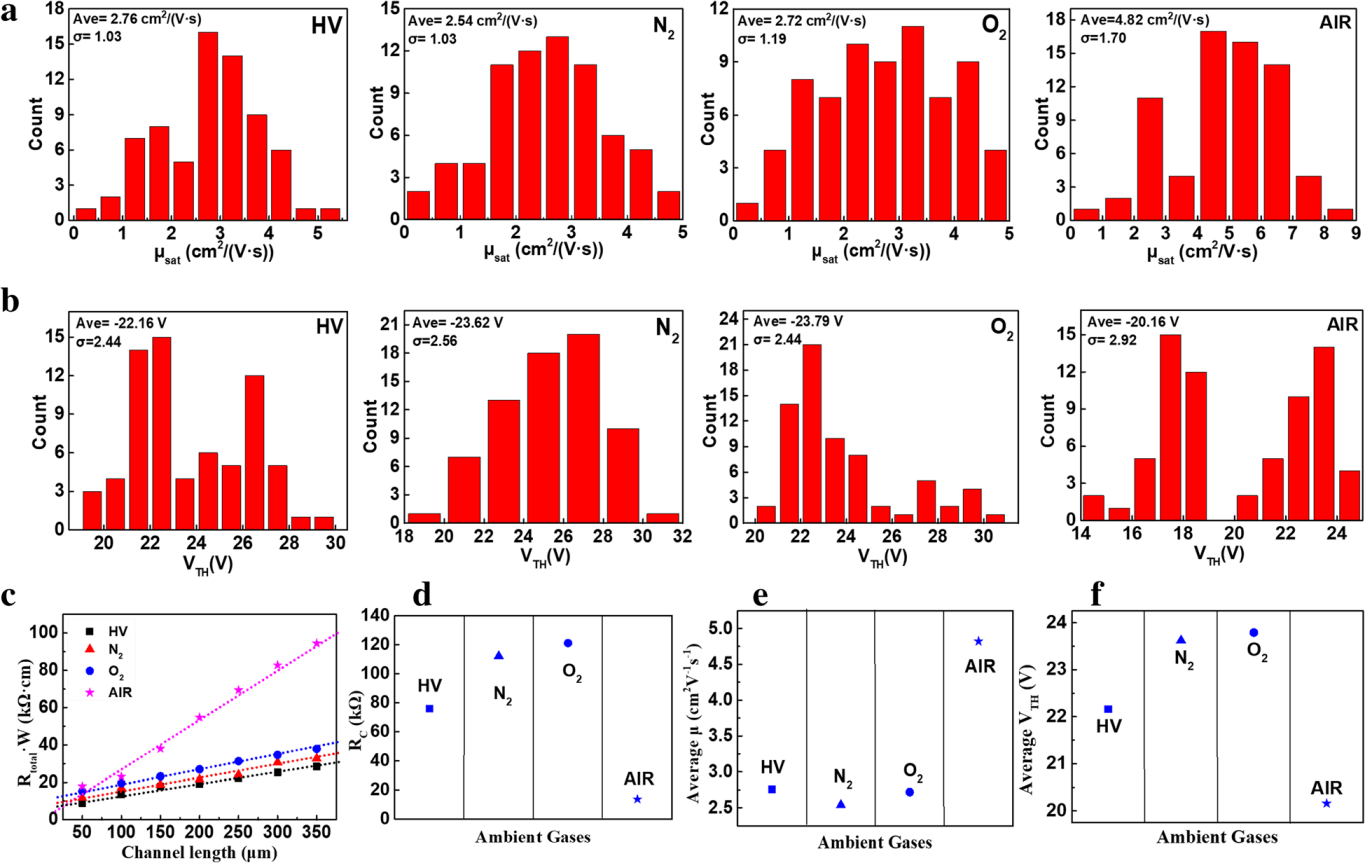
(Color online) Statistical histograms of the carrier mobilities (**a**) and threshold voltages (**b**) observed from devices exposed to various test gases. **c** Transmission line model plots with linear fittings of *R*_total_ *W* and impacts of environmental conditions on contact resistances (**d**), average mobilities (**e**), and average threshold voltages (**f**)

In order to get reliable and statistical data, we measured a total of 280 devices (70 devices for each environmental condition). The carrier mobility and threshold voltage experimental results are summarized and plotted as histograms in Fig. 3a and 3b. In addition, the average carrier mobilities, highest carrier mobilities, and average threshold voltages of devices exposed to various ambient gases are shown in Table 1. The highest average carrier mobility (4.82 cm^2^V^-1^s^-1^) and lowest threshold voltage (− 20.16 V) are observed with devices exposed to air. Thus, air-exposed devices exhibit the best electrical performances of the device types tested. The HV device, N_2_ device, and O_2_ device histograms indicate only slight differences in average carrier mobility, highest carrier mobility, and threshold voltage. It is known that air is composed of nitrogen (78%), oxygen (21%), moisture, etc. The HV, N_2_, and O_2_ devices exhibit similar electrical characteristics, which indicates that exposure to N_2_ and O_2_ does not produce significant performance differences relative to an HV device. One can assume that moisture plays a key role in improving the electrical performance of the air device. The relative humidity range during these experiments was 40–59%. Consequently, it is likely that H_2_O in the air affects device performance.

**Table 1:** Average and highest carrier mobilities, average threshold voltages, and *R*_C_ values of devices exposed to various gases

	HV	N_2_	O_2_	Air
Ave. mobility (cm^2^/V s)	2.76	2.54	2.72	4.82
Highest mobility (cm^2^/V s)	4.70	4.84	4.88	8.07
Ave. threshold voltage (V)	− 22.16	− 23.62	− 23.79	− 20.16
*R*_C_ (kΩ)	76	112.08	121.08	13.75

In order to understand gas exposure-based variation in the electrical properties of these C8-BTBT-based transistors, we measured *I*_D_-*V*_G_ curves of devices with channel lengths of 50 to 350 μm. Metal/semiconductor contact resistances (*R*_C_) were investigated for all four device types. We performed *R*_C_ extraction using the transfer line method, which is based on the following linear regime equation (1): [20].

1 $$ {\mathrm{R}}_{\mathrm{total}}={R}_{\mathrm{channel}}+{R}_{\mathrm{contact}}=\frac{L}{WC_i\left({V}_g-{V}_{\mathrm{th}}\right){\mu}_{\mathrm{channel}}}+{R}_{\mathrm{contact}} $$

Figure 3c shows the total resistances (*R*_total_) of devices exposed to different environmental conditions as functions of channel length. The *R*_C_ values are extracted from the *y*-intercepts of the fitting lines and plotted by exposure gas. *R*_C_ values are compared in Fig. 3d based on the results shown in Fig. 3c. Only small differences between the HV, N_2_, and O_2_ devices are noted. However, the air device exhibits a significant reduction in *R*_C_. The average carrier mobilities and average threshold voltages are summarized in Fig. 3e and 3f, respectively. The air devices exhibit much higher carrier mobilities and lower threshold voltages than their counterparts. The *R*_C_ values, average and highest carrier mobilities, and threshold voltages of the four device types are summarized in Table 1. Based on the results shown in Fig. 3d–f and Table 1, we can conclude that the improved electrical properties exhibited by the air devices are closely related to the reduced contact resistance between the C8-BTBT semiconductor and source/drain electrodes. Furthermore, the N_2_ and O_2_ device electrical properties do not deviate significantly from each other or those of the HV device. This indicates that the reduced *R*_C_ values that drive increased carrier mobilities and decreased threshold voltages are caused by H_2_O in air, rather than N_2_ or O_2_ concentrations. The mechanisms of this interaction are not clear, but we assume that hydronium and hydroxyl anions from H_2_O may passivate traps and defects in C8-BTBT semiconductors. Our present results provide further insights into the role of air in reducing contact resistances and improving overall electrical performance.

**Fig. 4:**
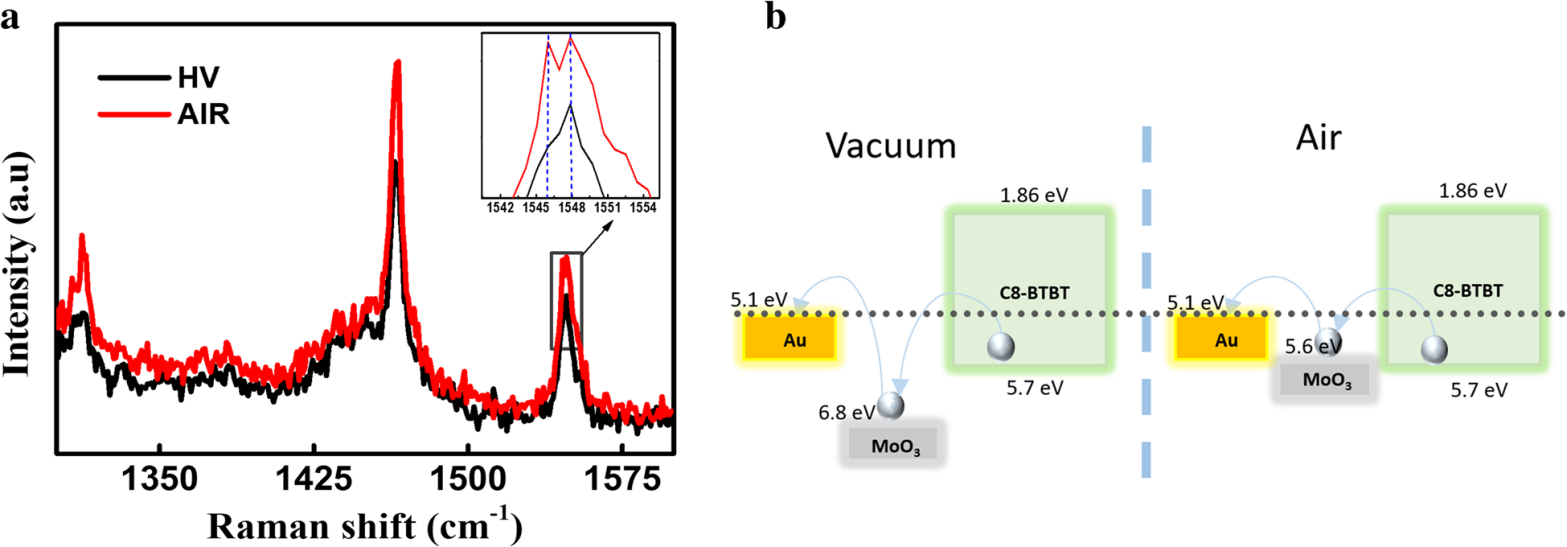
(Color online) (**a**) Raman spectra (*λ*_exc_ = 633 nm) of C8-BTBT thin films in HV and air conditions. The inset shows an enlargement of the area between 1542 and 1554 cm^−1^. (**b**) Schematic diagrams of work function changes in MoO_*x*_ in HV- and air devices, resulting in the reduction of the barrier height associated with charge injection from the S/D electrode to C8-BTBT

To further understand the mechanisms that drive differences in device electrical performance, we performed Raman spectra measurements of C8-BTBT films exposed to various environmental conditions. Figure 4a compares the Raman spectra of C8-BTBT films exposed to HV and air. Only the 1300 cm^−1^–1600 cm^−1^ spectral range is shown since these peaks are typically associated with C8-BTBT molecules and all of the charge sensitive bands lie in this region. Typically, C8-BTBT molecules orient themselves with the long-axis (*c*-axis) direction along the SiO_2_/Si substrate. A herringbone arrangement of BTBT core parts appears in the in-plane direction [14]. Thiophene peaks are located at 1314 cm^−1^ and 1465 cm^−1^, while the C–H in-plane peak appears at 1547 cm^−1^ [6, 21]. The Raman spectra of C8-BTBT samples exposed to HV, O_2_, and N_2_ do not exhibit significant differences. When the sample is exposed to air for a period of time, it exhibits Davydov splitting at 1547 cm^−1^ due to interactions between the hydroxyl anion from water and hydrogen from C–H groups. [22] The C–H bond from stacking of C8-BTBT molecules is typically suspended on the surface [14]. Thus, it can easily interact with moisture in the air and increase the carrier mobility via enhanced π-π and van der Waals interactions [5, 9]. This result provides further support for our previous assumption that hydroxyl anions passivate traps in the C8-BTBT films.

As Irfan et al. reported [23], the work function (*W*_F_) of the thermally evaporated 5.5 nm MoO_*x*_ is 6.82 eV. However, this decreases by 1.18 to 5.64 eV after 1 h of air exposure. The reduction in *W*_F_ upon air exposure may be due to moisture adsorption on the film surface. Based on the results shown by Irfan et al., we proposed a model that describes the effect of air exposure on C8-BTBT contact resistance and electrical performance (Fig. 4b) [9, 19, 23]. It is assumed that reducing the height of the contact barrier between the metal and the semiconductor would improve the carrier injection efficiency, reduce the contact resistance, and increase the carrier mobility. Another possible mechanism of *R*_C_ reduction is passivation of traps in the interface between C8-BTBT and the Au/MoO_3_ electrode. According to Wang et al., the metal/semiconductor interfacial trap density significantly affects the interfacial contact resistance [24]. In the present work, hydronium from water passivates interfacial traps, producing an *R*_C_ reduction.

**Fig. 5:**
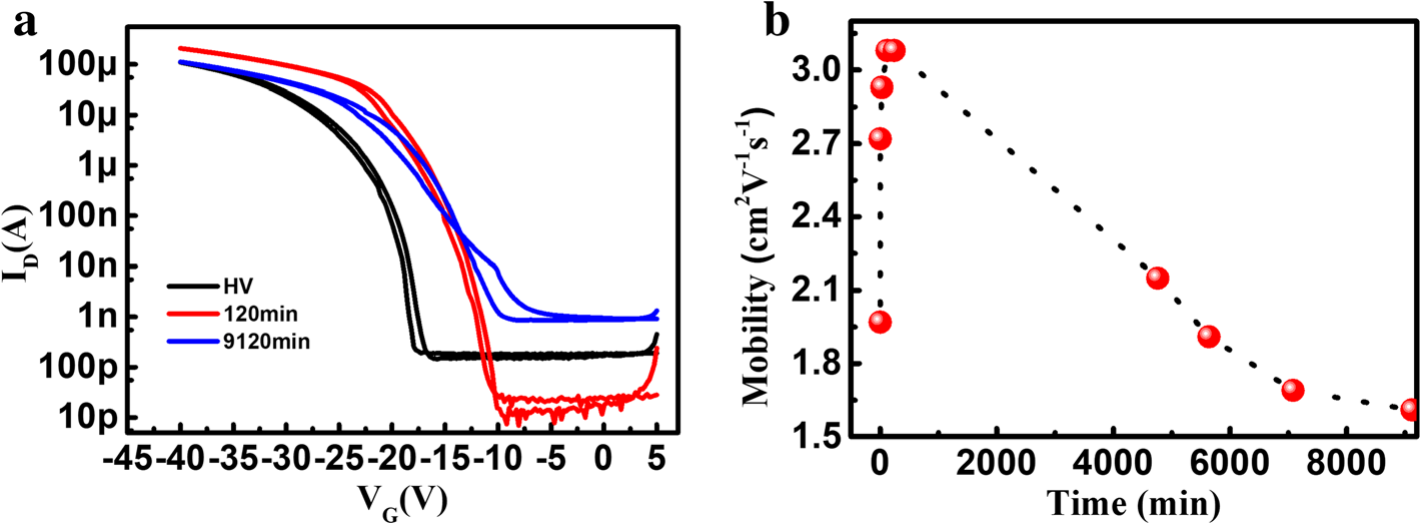
(Color online) (**a**) Typical *I*_d_-*V*_g_ characteristics of the HV device, 2-h air device, and 9120-min air device; (**b**) carrier mobility as a function of air-exposure time

Finally, the air stability of the C8-BTBT OTFTs was investigated. We measured the electrical properties of C8-BTBT devices that had been exposed to air for up to 9120 min (~ 1 week). Figure 5a compares *I*_D_-*V*_G_ characteristics of devices with air exposure times of 0 min, 2 h, and 9120 min. The carrier mobility is shown as a function of air exposure duration in Fig. 5b. The carrier mobility of a non-air exposed device is 1.97 cm^2^V^-1^s^-1^. The mobility increases with the air exposure duration until this duration reaches 4 h. The highest carrier mobility (3.08 cm^2^V^-1^s^-1^) is achieved after an air exposure time of 2 to 4 h. Further monitoring of the carrier mobility shows that it decreases gradually with additional air exposure. The carrier mobility decreases to 1.61 cm^2^V^-1^s^-1^ after the device has been exposed to air for 9120 mins (approximately 1 week). This carrier mobility degradation may occur because the channel is readily oxidized by moisture as shown below in Eq. (2) [25]. In this equation, OSC and OSC+ represent the organic semiconductor and molecular cation, respectively.

2 $$ 6{\mathrm{H}}_2\mathrm{O}+4{\mathrm{O}\mathrm{SC}}^{+}\rightleftharpoons 4\mathrm{OSC}+{\mathrm{O}}_2+4{\mathrm{H}}_3{\mathrm{O}}^{+} $$

After a period of air exposure, moisture adsorption induces unoccupied states above the HOMO and generates deep hole traps, which significantly degrade carrier transport in the channel and increase the contact resistance [24]. Gomes et al. and Peter et al. have demonstrated that water on the surface of SiO_2_ plays an important role in p-type OTFTs. Due to the Si–O–H ↔ Si–O^−^ + H^+^ reaction, a significant amount of hydronium is present in the absorbed water layer [26]. In addition, mobile charges in the semiconductor are slowly replaced by immobile charges at the SiO_2_ surface that can reversibly migrate into bulk SiO_2_. Therefore, exposure to air for a long time, constant absorption and interaction of moisture will lead to increased transistor instability [27] and reduce its carrier mobility.

Using a comparative study of devices exposed to various gas environments, we demonstrated that moisture in the air has a significant impact on the electrical performance characteristics of C8-BTBT-OTFT devices. We also found that an appropriate air-exposure time can improve device electrical performance but a long exposure time degrades it. It is widely believed that exposing organic devices to air is harmful to their electrical properties. The present work also demonstrates the positive role of moisture in passivating C8-BTBT semiconductor traps and lowering *R*_C_ values. It also provides useful insights into the ideas that may improve C8-BTBT OTFT device performance and improve knowledge of their air stability.

## Conclusions

In summary, we have investigated the effects of ambient gases on the electrical properties of solution-processed C8-BTBT OTFTs. The electrical properties of devices exposed to various ambient gases (HV, O_2_, N_2_, and air) were compared. We observed that the electrical properties of the O_2_ device and N_2_ device varied little relative to the HV device. However, a significant improvement in electrical properties was observed with the air device. For the 70 devices with 2 h of air exposure, the average and highest carrier mobilities were 4.82 and 8.07 cm^2^V^-1^s^-1^, respectively. This compares to 2.76 and 4.70 cm^2^V^-1^s^-1^ for HV devices. The lowest threshold voltages were also observed using the air devices. The improved electrical performance of the air device is thought to be due to reduced contact resistance and decreased MoO_3_ work function after air exposure. In addition, C8-BTBT OTFT air-stability was investigated. The electrical performance degraded upon exposure to air for more than 4 h. This work provides a systematic understanding of the influence of environmental conditions on the electrical performance characteristics of solution processed C8-BTBT OTFTs. It aids in the development of high performance, air-stable, printable OTFT devices.

## Abbreviations

AFM: Atomic force microscopy
Au: Gold
C8-BTBT: 2,7-dioctyl [1] benzothieno[3,2-b][1]-benzothiophene
HOMO: Highest occupied molecular orbital
HV: High vacuum
*I*_D_: Drain current
L: Channel length
MoO_3_: Molybdenum oxide
OTFTs/OTFT: Organic thin-film transistors
PMMA: Polymethyl methacrylate
*R*_C_: Contact resistance
RMS: Root mean square
*R*_total_: Total resistances
TFTs: Thin-film transistors
*V*_G_: Gate voltage
*W*: Channel width
